# The effect of bacterial cellulose nanocrystals on the shear bond strength of resin modified glass ionomer cement to dentin

**DOI:** 10.4317/jced.58153

**Published:** 2021-08-01

**Authors:** Marzieh Moradian, Dana Jafarpour, Maryam Saadat, Farzin Tahmasebi

**Affiliations:** 1 Department of Operative Dentistry, School of Dentistry, Shiraz University of Medical Sciences, Shiraz, Iran; 2 Biomaterials Research Center, School of Dentistry, Shiraz University of Medical Sciences, Shiraz, Iran; 3 Student Research Committee, School of Dentistry, Shiraz University of Medical Sciences, Shiraz, Iran

## Abstract

**Background:**

The present study aimed to investigate the effect of bacterial cellulose nanocrystals (BCNC) on the shear bond strength (SBS) of resin modified glass ionomer cement (RMGIC) to dentin.

**Material and Methods:**

A total of 48 freshly extracted intact third molars were randomly divided into four main groups with three different concentrations (0.3%, 0.5% and 1% wt) of BCNC with RMGIC and control group without BCNC. These specimens were kept in distilled water at 37° C for 24h. Shear bond strength was examined, using the universal testing machine. Kruskal-Wallis test and Dunn`s post-hoc test were applied for analysis of data. *P*<0.05 was considered as the level of significance.

**Results:**

The addition of a 1%wt of BCNC to the RMGIC led to a significant increase in the shear bond strength (7.17 ± 2.14) compared to the control group (2.09 ± 1.80) (*P*=0.007). The shear bond strength was improved up to 343%.

**Conclusions:**

It was found that the incorporation of 1% wt BCNC to the RMGICs enhanced the SBS properties of the RMGIC significantly. Modifying RMGIC with BCNC might be advantageous in terms of improving the restorative material.

** Key words:**Bacterial cellulose nanocrystals, RMGIC, Shear bond strength.

## Introduction

Glass ionomer cements (GICs) are extensively used as luting, base, liners and restorative materials worldwide due to their promising features such as chemical adhesion (1), biocompatibility (2), and fluoride release (3). However, due to low mechanical properties, their application has been limited to non-load-bearing areas. The development of RMGIC with the incorporation of bisphenol-glycidyl-methacrylate (Bis-GMA) or hydroxyethyl-methacrylate (HEMA) to the conventional glass ionomer cements led to improved mechanical properties and resistance against bacterial adhesion and reduced solubility (4). However, still some weaknesses in the bond strength and mechanical properties of this material need to be overcome. Various modifications such as the addition of filler components, such as glass fiber, hydroxyapatite (5), bioactive glass particles (6), and silver-amalgam particles (7) have been developed with an aim to augment GIC properties. Although several attempts have been made to enhance GICs properties through the addition of these reinforcements (8-10), the extensive application of these components has been stalled due to its numerous downsides. To name a shortcoming, it has been reported that fillers can impede the glass ionomer cement antibacterial properties (11).

Recently, the application of nanoparticles has gained widespread attraction in dentistry since they increase the mechanical properties and at the same time maintain an antibacterial effect (12). Cellulose nanocrystals (CNCs) are highly crystalline rod-like structures with nanoscale dimension which are obtained from cellulose and offer superb mechanical properties. Bacterial cellulose, which is primarily created by members of the Gluconacetobactergenus, is a favorable starting material for extracting CNCs due to its high purity and crystallinity. Bacterial CNC (BCNC) has appealed great attention since it presents excellent characteristics such as high capacity for water retention, increased strength in wet phase, biodegradability, as well as biocompatibility. These properties make BCNC a promising material for medical fields.

Previously, some studies have claimed that the addition of cellulosic fibers might enhance the mechanical properties of restorative materials. Silva *et al*., in their study assessing the effect of cellulosic fibers on the mechanical strength of restorative materials, reported that the cellulosic fiber-modified glass ionomer cement showed increased compressive strength and abrasion resistance and higher bond strength to dental structures (13).

Enamel adhesion has become a routine and reliable aspect of modern restorative dentistry. However, the adhesion of dental materials to dentin is more challenging and less predicTable, due to its intricate histological composition. GIC bonds chemically to enamel as well as to dentin (14,15). As one of the mechanical properties of GICs, the bond strength of GIC is a determinative factor in the clinical durability of these restorations. Shear bond test, which is used to inspect the shear bond strength of dental materials, plays an important role in refining the materials function against masticatory forces (16). *In vitro* bond strength tests are valuable in terms of evaluating the materials probable correlation with clinical issues (17). The bond strength between dentin and the RMGIC is an important criteria contributing to the success of these restorations. Since no study has been previously conducted to evaluate the effect of BCNC on the bond strength of dentin to glass ionomer cements, the current study was carried out to inspect the incorporation of BCNC on shear bond strength of RMGIC to dentin. The null hypothesis was that the SBS did not differ between RMGIC and BCNC-incorporated RMGIC.

## Material and Methods

For this *in vitro* study, bacterial cellulose nanocrystals (Gorgan Nanonvin Polymer Co., Iran) were used. Bacterial cellulose was extracted from Gluconacetobactergenus.

Forty-eight extracted human third molars were collected and tested within six months after extraction. The selected teeth were devoid of any caries, restoration, and cracks. Residual soft tissue was removed carefully, and teeth were kept in distilled water with a 0.1% thymol as a disinfectant at 4°C for 1 week, and then remained in distilled water at 37°C until tested. Teeth were mounted at 2mm below the cement enamel junction (CEJ), in self-polymerizing acrylic resin (Acropars, Iran) in a rectangular shaped epoxy resin mold (30×20×15mm) as their occlusal portion were available for bonding.

The occlusal surfaces of the teeth were transversally sectioned by diamond disk (D&Z, Germany) to expose the superficial dentin just beneath the dentinoenamel junction (DEJ). The procedure was conducted under running water to cool the teeth. The exposed dentin surfaces of the specimens were wet grounded with 1000 grit silicon carbide abrasive papers to achieve homogenous surface. Then the dentin surface was conditioned with dentin conditioner polyacrylic acid 10% (GC, Tokyo, Japan) for 20 seconds by means of a micro brush, then were rinsed by distilled water for 20 seconds, and dried by cotton pellets.

To form the control group, resin modified glass ionomer cement powder (Fuji II LC GC, Tokyo, Japan) was mixed with its liquid using a liquid to powder ratio of 1g: 3.2g (two drops of liquid and one module of modified powder) without the addition of bacterial cellulose nanocrystals.

For other experimental groups, the specimens were divided into three groups (n=12) in accordance with the bacterial cellulose nanocrystal weight percentage incorporation to RMGIC: glass powder containing 0.3, 0.5, and 1 wt% bacterial cellulose nanocrystal were respectively mixed with resin-modified GIC liquid with a liquid to powder ratio of 1g: 3.2g (two drops of liquid and one module of powder). Using an electronic balance (GR-3000, A & D CL Toshiba, Tokyo, Japan), Bacterial nanocrystalline powders were weighed carefully to an accuracy of 0.001 g and were added to the previously weighed glass powder according to their respective group ratio (0.3, 0.5, and 1 wt%). In order to obtain a homogenous dissemination of particles, BCNC and glass powders were initially mixed by hand for 15 seconds and then were blended with amalgamator (Ultramat 2; SDI, Australia) for 20 seconds.

The prepared pastes were placed in cylindrical silicon mold (3 mm diameter and 2 mm height) on center of superficial dentin and a Mylar strip was located on the top surface of the mold. The specimens were light cured for 40 seconds to warrant a perfect setting by using a light emitting diode (LED) polymerizing unit (Demi Plus; Kerr, Switzerland) at an intensity of 1200 mW/cm2.

After setting, the silicon molds were removed. The bonded specimens were painted over with varnish (Kimia, Iran) and teeth were preserved in distilled water until exposed to SBS test.

**Figure 1:**
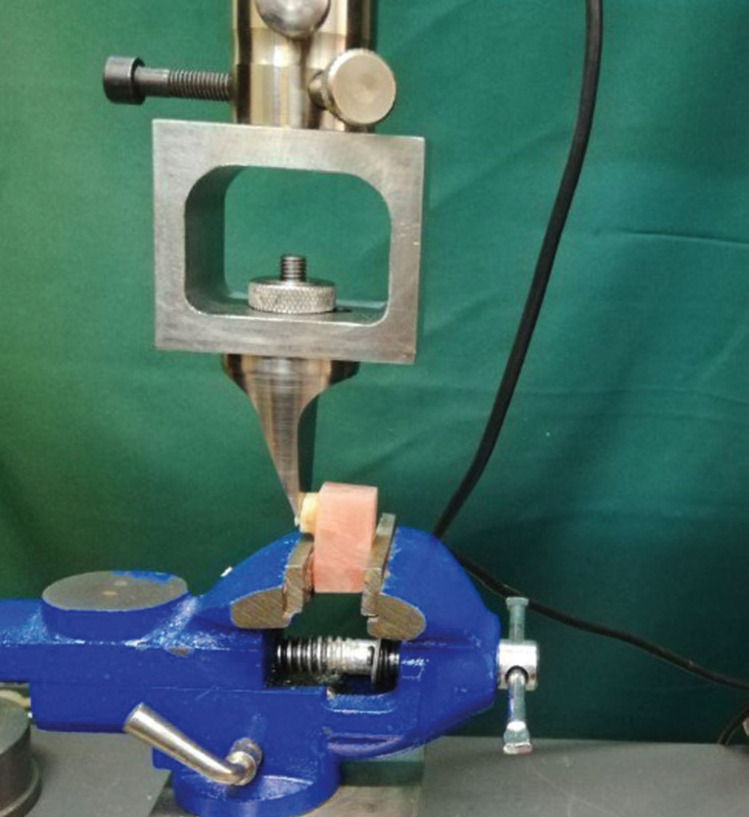
The shear bond strength of each specimen was tested by a steel wedge-shaped blade and crosshead speed of 0.5 mm/minute using a universal testing machine.

Using a universal testing machine (ZwickRoell, Germany), the shear bond strength of each specimen was tested by a steel wedge-shaped blade and crosshead speed of 0.5 mm/minute (Fig. 1). SBS values were measured and reported in MPa.

-Statistical analyses

The obtained SBS value were analyzed with SPSS software (version 25) using Kruskal-Wallies H test and Dunn`s post-hoc test at significance level of *p*<0.05.

## Results

The mean SBS values of the test groups have been shown in Table 1. As revealed in Table 1, the control group had the lowest shear bond strength (2.09 ± 1.80) among study groups. The addition of BCNC led to an increase in the shear bond strength of all study groups with the highest increase in group IV (1% wt) (7.17 ± 2.14). In conformity with Kruskal-Wallis H test, the shear bond strength values for the study groups were as follows: Group IV (1% wt)>Group III (0.5%) > Group II (0.3%)> Group I (control).

**Table 1:**
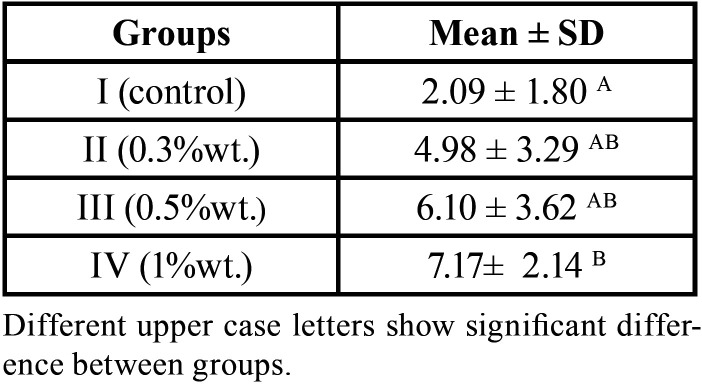
Mean ± SD shear bond strength (MPa) of the experimental groups.

According to Dunn`s post-hoc test, there was a significant difference in SBS values between group IV (1%wt) and the control group (*P* value= 0.007). However, no statically significant difference in SBS was detected with different concentrations of BCNC (*P*>0.05).

**Figure 2:**
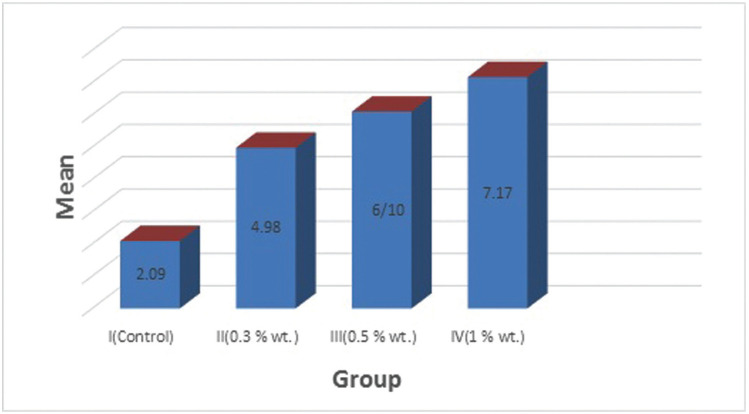
.Comparison of mean SBS values (MPa) between study groups.

As shown in Figure 2, group IV (1%wt) had the highest SBS and the control group showed the lowest mean shear bond strength among the study groups.

## Discussion

To the authors’ best knowledge, the present research was the first to inspect the effect of bacterial cellulose nanocrystals on the SBS of RMGICs. The null hypothesis was rejected as the findings revealed that BCNC-incorporated RMGIC had greater SBS to dentin compared with the control group.

Glass ionomer cement is regarded as an imperative revolutionary in restorative dentistry, due to its chemical adhesion to tooth, coefficient of thermal expansion close to tooth structures, biocompatibility, and fluoride release. Yet, this cement present some drawbacks with regards to mechanical integrity and load bearing capacity (18). Previous literature has verified significantly greater mechanical and bond strength for RMGICs compared to the conventional GIC. Still, low mechanical properties limit the application of these restorative materials to low load bearing sites including Class III and Class V cavities (19). In modern dentistry, the application of nanotechnology has become enormously established. The addition of nanoparticles to the cement matrix is an innovative approach which has aimed to ameliorate the mechanical strength of glass ionomer cements (20).

Previously, some studies assessed the effects of incorporating various nanoparticles into GICs on their mechanical properties. Kheur *et al*. showed that the addition of 6% wt Hydroxyl Apatite particles in the range of 80–150 nm significantly improved the shear bond and flexural strength of a conventional GIC at a liquid to powder ratio of 3:1 (21). In another work by Jowkar *et al*. (22) the authors revealed that GIC comprising 0.1 and 0.2 wt % silver nanoparticles had significantly greater surface microhardness, compressive strength, and microshear bond strength to dentin compared with the non-reinforced glass ionomer cement. In line with those studies, our findings revealed that the addition of bacterial cellulose nanocrystals into RMGIC led to in an increase in the shear bond strength of all of the test groups.

Cellulosic extractions have been studied for several years as reinforcing agents to be added into materials, particularly polymers. Obtained from fiber, cellulose nanocrystals are crystalline spheres produced by means of acid hydrolysis and bacteria (23).

Due to its high purity and crystallinity, bacterial cellulose, which is primarily created by members of the Gluconacetobactergenus, is an ideal starting material to extract CNCs. High capacity for water retention, increased strength in wet phase, biodegradability, as well as biocompatibility are among the favorable features of BCNCs. The mechanical strength of the GIC modified with cellulosic fibers has been proven to enhance, having a direct relation with the fiber concentration. However, no previous study investigated the effect of bacterial CNC on those properties. Therefore, the present study sought to assess the bond strength of RMGIC reinforced with BCNC.

GIC bonds chemically to enamel as well as to dentin (14). Bonding to normal dentin is a greater challenge when compared with the enamel, owing to its organic composition and complex structure (24). Therefore, in this study, BCNC was added into RMGIC in an attempt to augment the dentin-glass bond strength. The bond strength was measured using the shear bond test. The shear bond strength test arrangement is the most common laboratory technique to evaluate the performance of the bonded restorations, especially in GIC, which deal with inferior bond strength (25).

Based on ISO 9917-1:2007 instructions, any alteration in the liquid to powder ratio of the GIC might influence the final clinical properties of the material (26). Hence, finding an optimum proportion in which BCNC could be added to GICs is essential. According to Silva *et al*., the addition of CNC fibers with a concentration above 1% lead to the composites mechanical failure due to the aggregation of nanoparticles. It has been reported that owing to the specificity of the nanomaterials, extremely low concentrations are needed for material augmentation (18).

Thus, in the present study, the effect of bacterial cellulose nanocrystals was evaluated in three weight percentages of 0.3%, 0.5%, and 1%. According to the findings, the addition of 0.3%, 0.5%, and 1% wt BCNC to RMGIC led to an increase in bond strength of RMGIC to dentin. However, only the RMGIC containing 1% wt BCNC represented significantly superior shear bond strength compared to the control group.

In line with this finding, previous studies have substantiated an improvement in GIC strength following the addition of cellulosic derivatives. Jing sun *et al*. revealed the profit of the co-doped of CNC and TiO2 nanoparticles could increase the mechanical properties of GIC. The researchers found that the composition of CNC and TiO2 increased compressive strength and shear bond strength (20).

A possible justification for the superior shear bond strength of the BCNC incorporated GIC could be the fact that the small sized nanocrystals might lodge in the vacant sites amid the larger glass particles, offering an extra bonding spot for the polyacrylic polymer, which might eventually lead to GIC reinforcement. Another eventual reason for increasing SBS in RMGIC containing BCNC might be the fact that BCNC swelling with the polyacrylic acid solution escalates the nanocrystals reactivity within the GIC matrix and tooth assembly. This phenomenon might perhaps upturn the chemical bonds between the calcium ions and phosphate present in dentin and the hydroxyl groups existing in the nanocrystals.

It is noteworthy to mention that incorporating BCNC to the RMGIC matrix was a simple and practical method as the bacterial cellulose nanocrystals homogeneously disseminated within the resin-modified glass ionomer cement matrix without compromising RMGIC matrix fluidity.

The results of this *in vitro* study cannot be generalized to clinical settings. For this reason, further *in vivo* studies are required to confirm the findings of the present study. Moreover, in this study, drained amalgam capsules were used for the preparation of a homogenous mixture of RMGIC-BCNC. Therefore, it is recommended that in order to reach a homogenous powder mixture, capsulated GICs be tested using specific GIC mixers. Furthermore, any potential alteration in RMGIC long-term bond strength and antibacterial activity following BCNC addition needs to be further studied.

## Conclusions

Within the limitations of this study, it can be concluded that the addition of BCNC into RMGIC resulted in an increase in the shear bond strength of the material. The increase in the shear bond strength of RMGIC was statistically significant only when mixed with 1 wt% BCNC. Modifying RMGIC with BCNC might be advantageous in terms of improving the restorative material.
